# Identification of *TMEM129*, encoding a ubiquitin-protein ligase, as an effector gene of osteoarthritis genetic risk

**DOI:** 10.1186/s13075-022-02882-y

**Published:** 2022-08-08

**Authors:** Abby Brumwell, Guillaume Aubourg, Juhel Hussain, Eleanor Parker, David J. Deehan, Sarah J. Rice, John Loughlin

**Affiliations:** 1 Newcastle University, Biosciences Institute, International Centre for Life, Newcastle upon Tyne, UK; 2 Freeman Hospital, Newcastle University Teaching Hospitals NHS Trust, Newcastle upon Tyne, UK

**Keywords:** Genetics, Epigenetics, DNA methylation, Epigenome editing, *TMEM129*

## Abstract

**Background:**

Osteoarthritis is highly heritable and genome-wide studies have identified single nucleotide polymorphisms (SNPs) associated with the disease. One such locus is marked by SNP rs11732213 (T > C). Genotype at rs11732213 correlates with the methylation levels of nearby CpG dinucleotides (CpGs), forming a methylation quantitative trait locus (mQTL). This study investigated the regulatory activity of the CpGs to identify a target gene of the locus.

**Methods:**

Nucleic acids were extracted from the articular cartilage of osteoarthritis patients. Samples were genotyped, and DNA methylation was quantified by pyrosequencing at 14 CpGs within a 259-bp interval. CpGs were tested for enhancer effects in immortalised chondrocytes using a reporter gene assay. DNA methylation at the locus was altered using targeted epigenome editing, with the impact on gene expression determined using quantitative polymerase chain reaction.

**Results:**

rs11732213 genotype correlated with DNA methylation at nine CpGs, which formed a differentially methylated region (DMR), with the osteoarthritis risk allele T corresponding to reduced levels of methylation. The DMR acted as an enhancer and demethylation of the CpGs altered expression of *TMEM129*. Allelic imbalance in *TMEM129* expression was identified in cartilage, with under-expression of the risk allele.

**Conclusions:**

*TMEM129* is a target of osteoarthritis genetic risk at this locus. Genotype at rs11732213 impacts DNA methylation at the enhancer, which, in turn, modulates *TMEM129* expression. *TMEM129* encodes an enzyme involved in protein degradation within the endoplasmic reticulum, a process previously implicated in osteoarthritis. *TMEM129* is a compelling osteoarthritis susceptibility target.

**Supplementary Information:**

The online version contains supplementary material available at 10.1186/s13075-022-02882-y.

## Background

Primary osteoarthritis (OA) is highly polygenic, with single nucleotide polymorphisms (SNPs) acting cumulatively to increase disease risk [1]. OA-associated SNPs are detected via genome-wide association studies (GWAS) undertaken on large case-control cohorts [1]. In 2019, a GWAS of hip and knee OA was performed utilising the UK Biobank and arcOGEN datasets, encompassing 77,052 cases and 378,169 controls [2]. This study identified 64 OA risk signals, 52 of which were novel [2].

Transitioning from a genetic association signal to the gene targeted by that signal - the effector gene - is a critical step in the clinical exploitation of GWAS data [3]. Many risk-conferring alleles for common complex diseases reside within gene-regulatory elements such as enhancers, with their deleterious effect being a change in the expression of a nearby gene through altered enhancer activity [4, 5].

Our analysis of the novel OA signals from the 2019 UK Biobank and arcOGEN study revealed that genotype at the associated SNPs often correlated with the level of methylation of nearby CpG dinucleotides in cartilage DNA, forming *cis* methylation quantitative trait loci (*cis*-mQTLs) [6]. We hypothesise that these mQTLs are functional intermediates between genotype and OA risk; the risk-conferring allele alters methylation at CpGs, which modulates the binding of transcription factors and the expression of a nearby target gene, increasing disease risk [7–11]. If such links exist, they may be amenable to intervention as therapeutic targets [12]. By combining in silico and experimental approaches, we have begun to generate data supporting this functional link between OA genetic signals, DNA methylation, and target gene expression [13–16]. In this report, we focus on the analysis of one of the signals from the UK Biobank and arcOGEN study, marked by SNP rs11732213 [2].

This T > C SNP lies within an intron of *SLBP* on chromosome 4p16.3 and correlates with hip and knee OA, with *P*-value of 8.81×10^−10^ and odds ratio of 1.06 [2]. T is the major and OA risk-conferring allele, with a frequency of 0.83 in European ancestry cohorts. By using the Illumina 450K CpG methylation array, we discovered that genotype at the SNP correlates with the methylation of two CpGs in cartilage DNA, cg20987369 and cg25007799 [6]. These CpGs are only 85bp apart and are located 125kb from rs11732213, with the risk-conferring allele T of the SNP correlating with lower methylation at both CpGs [6]. The CpGs reside adjacent to a predicted transcriptional regulatory element [6]. The physical closeness of the two CpGs combined with their location near to a potential regulatory element increases the likelihood that this is a genuine OA mQTL. We therefore prioritised this signal for detailed further investigation.

We have used patient cartilage samples, a human chondrocyte cell line, and a range of molecular approaches including epigenome editing to investigate and characterise this rs11732213 OA mQTL and to identify target gene/s of the association signal.

## Methods

### In silico analysis

The UCSC genome browser [17] was used to visualise the genomic area encompassing rs11732213, cg20987369 and cg25007799 and to highlight genes within the interval. Chromatin state data from the ROADMAP epigenomics project [18] was used to identify areas of regulatory function or active transcription. The Washington University epigenome browser’s ChIA-PET and Hi-C data [19] were used to investigate long-range chromatin interactions in all available cell line and primary cell data. JASPAR [20] was used to search for transcription factors that bound at or close to CpGs of interest. The expression of transcription factors in cartilage was assessed using RNA-sequencing data generated from the hip cartilage of ten OA and six neck of femur (NOF) fracture patients ([21]; Gene Expression Omnibus (GEO; https://www.ncbi.nlm.nih.gov/geo/) accession number GSE111358).

### Patient samples

Cartilage samples were obtained from 165 patients undergoing joint replacement surgery at the Newcastle upon Tyne NHS Foundation Trust hospitals for primary hip (*n* = 65) or knee (*n* = 100) OA. Patient details are available in Supplementary Table 1. Cartilage samples were processed, and the nucleic acids were extracted as previously described [14–16].

### SNP genotyping

Pyrosequencing assays were designed using PyroMark Assay Design Software 2.0 (Qiagen) and ordered from Integrated DNA Technologies (IDT). Genomic DNA encompassing the SNP was amplified by PCR and genotype was assessed using the PyroMark Q24 Platform (Qiagen) according to the manufacturer’s instructions [16]. Oligonucleotide primer sequences are available in Supplementary Table 2.

### DNA methylation analysis

Five pyrosequencing assays were designed to capture 14 CpGs, spanning a 259bp region and encompassing cg20987369 and cg25007799. Genomic DNA (500ng) was bisulphite converted using the EZ DNA methylation kit (Zymo Research). Bisulphite-converted DNA was then amplified using PCR and methylation quantified using the PyroMark Q24 Platform (Qiagen). Analysis was performed in duplicate and replicates that exceeded a 5% variance were excluded from analysis for quality control purposes. Primer sequences are available in Supplementary Table 2.

### Lucia reporter assay

A 568bp region encompassing the 14 CpGs was amplified using genomic DNA (primer sequences in Supplementary Table 2), then cloned into the pCR-Blunt-II-TOPO vector (Invitrogen) and transformed into chemically competent *E. coli*. Colonies were grown overnight; plasmid DNA was extracted, and Sanger sequenced (Source Bioscience). Plasmids containing the desired insert were digested using the restriction enzymes *AvrII* and *SpeI* (New England Biolabs). The insert was cloned into a pCpGfree-promoter-Lucia plasmid (InvivoGen), then methylated and mock-methylated using *M.SssI* (New England Biolabs). Tc28a2 immortalised chondrocytes [22] were seeded onto a 96-well plate at 5000 cells/well and transfected 24h later with 100ng pCpG-free-promoter DNA and 10ng pGL3-promoter (Promega) with Lipofectamine 2000 (Invitrogen). Cells were lysed after 24h and luminescence was read using the Dual-Luciferase Reporter Assay System (Promega) [15]. Six biological replicates were performed for each plasmid construct.

### Epigenome modulation using dead Cas9 (dCas9)

Three guide RNA sequences (gRNAs 1–3; Supplementary Table 3) were designed within the region encompassing CpGs1-14, using the IDT gRNA design tool. Demethylation of the region was conducted using Tc28a2 cells that stably express an inducible dCas9-TET1 construct [14]. Guide RNAs were diluted, annealed with *trans*-activating CRISPR RNA (tracrRNA) and transfected into Tc28a2/dCas9-TET1 as previously described [14]. Controls expressed dCas9-TET1 and tracrRNA but had a non-targeting gRNA (Alt-R CRISPR-Cas9 negative control crRNA, IDT product number 1072544). Cells were grown in the transfection mix for 24h and then expanded into T25 flasks with complete growth media. Cells were harvested 72h post-transfection. Nucleic acids were extracted as previously described [16]. Twelve biological replicates were performed for each condition.

### Quantitative gene expression

cDNA was reverse transcribed using Superscript IV (Invitrogen) with an input of 1μg total RNA. Gene expression was measured by real-time quantitative PCR (RT-qPCR) using pre-designed TaqMan assays (IDT; Supplementary Table 4). Genes of interest were normalised to housekeeping genes *18S*, *GAPDH* and *HPRT1*. Gene expression was determined using the 2^-ΔCt^ method as previously described [23].

### Allelic expression imbalance (AEI)

Transcript SNPs within exons of *TMEM129* (rs2236786) and *SLBP* (rs2247341) were identified, and AEI was quantified by pyrosequencing using the PyroMark Q24 Platform (Qiagen) [16]. Matched genomic DNA and cDNA from patients who are compound heterozygous at rs11732213 and the transcript SNP for the investigated gene were used for analysis. Allelic expression of the cDNA was normalised to its respective genomic DNA. Samples were analysed in triplicate and replicates that exceeded a 5% variance were excluded from the analysis. Pairwise *r*^2^ and D′ values in European ancestry cohorts were determined using LDlink (https://ldlink.nci.nih.gov). Primer sequences are available in Supplementary Table 2.

### Statistical analyses

For graphical representations of DNA methylation data, methylation status was plotted in the form of *β*-values, ranging from 0 (no methylation) to 1 (100% methylation). For statistical analysis of methylation data, *β*-values were converted to M-values [24]. In mQTL analysis, linear regression was used to assess the relationship between CpG methylation and genotype at rs11732213. When the number of rs11732213 minor allele homozygotes (CC) was three or more, all three genotype groups were plotted and analysed. When the number of minor allele homozygotes was less than three, the minor allele homozygotes were combined with the heterozygotes (TC + CC). Mann-Whitney *U* test was used to compare DNA methylation levels following stratification by joint site or sex irrespective of genotype. Lucia reporter data was analysed using an unpaired *t*-test. Changes in gene expression following dCas9-TET1 modulation were analysed using a paired *t*-test with the Holm-Šídák approach to account for multiple comparisons. Wilcoxon matched-pairs signed rank test was used for AEI analysis. Tests were performed in GraphPad Prism.

## Results

### Physical interactions between OA-associated CpGs and putative effector genes

**Fig. 1:**
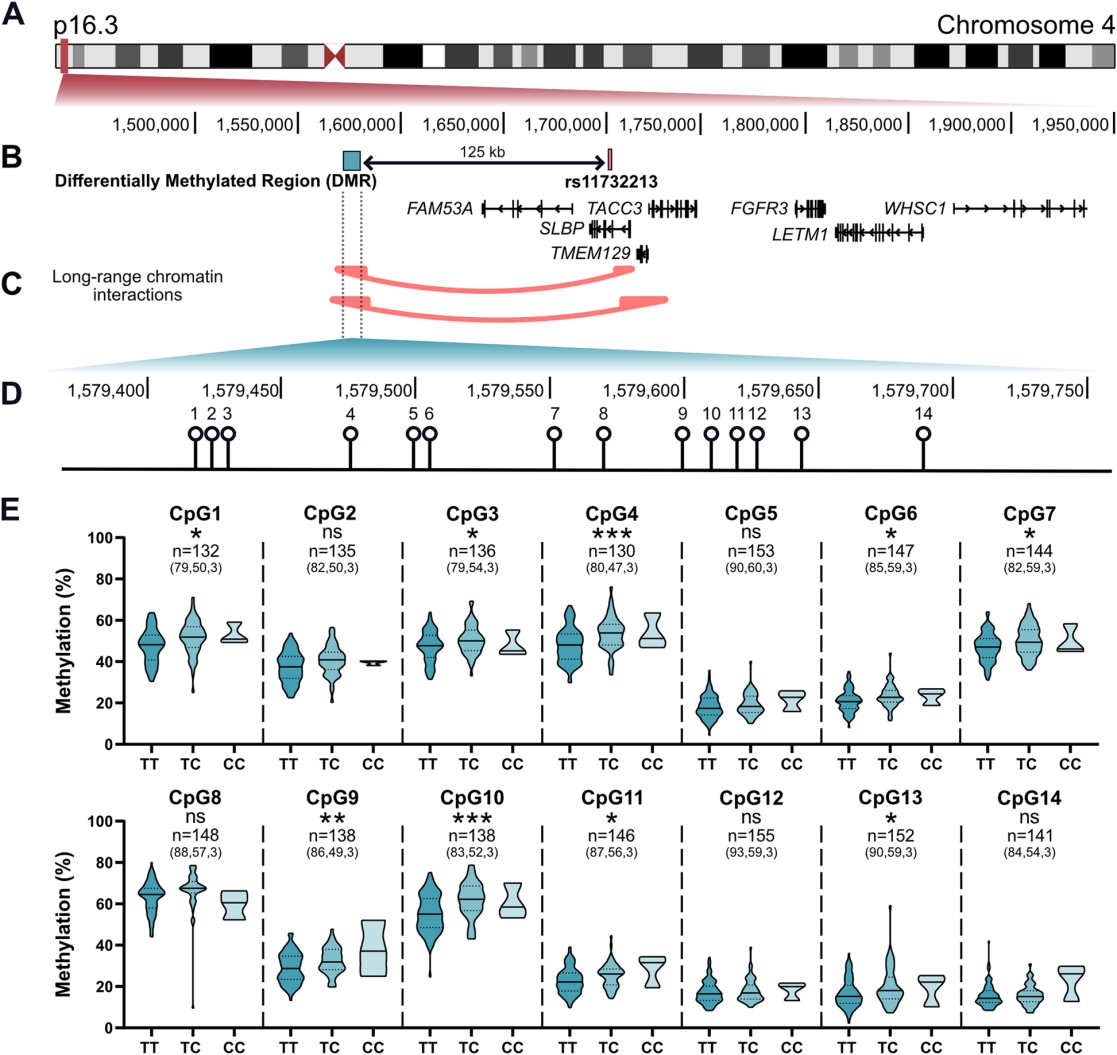
In silico and methylation quantitative trait locus (mQTL) analysis of the rs11732213 OA risk signal. **A** Schematic representation of chromosome 4 highlighting in red the physical location of the signal. Coordinates from UCSC hg19. **B** Schematic localisation of rs11732213, of cg20987369 and cg25007799 (within the DMR), and of all nearby genes at the signal. **C** Schematic of long-range chromatin interaction from ChIA-PET in the K562 cell line (top red curve) and from Hi-C in the IMR-90 and H1-hESC cell lines (bottom red curve) sourced from the Washington University epigenome browser. **D** Magnification of the DMR and the position of all 14 CpG sites in the region. cg20987369 is CpG8, cg25007799 is CpG13. **E** mQTL analysis showing cartilage DNA methylation values at the CpGs according to genotype at rs11732213. Methylation data is in the form of *β*-values ranging from 0 (no methylation) to 1 (complete methylation) and expressed as a percentage. In the violin plots, solid and dashed horizontal lines represent the median and interquartile range. Difference in numbers (*n*) due to variable number of patient samples per CpG passing quality control, with numbers in parentheses the number of patients per genotype (TT, TC, CC). *P*-values calculated by linear regression. * = *P* < 0.05; ** = *P* < 0.01; *** = *P* < 0.001; ns = not significant (*P* > 0.05)

Using in silico data [17–19] we searched for physical interactions across the association signal marked by rs11732213, encompassing putative effector genes, and the CpGs of interest (cg20987369 and cg25007799) (Fig. 1A–C). Using a ChIA-PET dataset [19] we observed an interaction between the CpGs and a region including the 5′ end of *SLBP* and sequence downstream of *TMEM129* in K562 cells, an immortalised myelogenous leukaemia cell line (Fig. 1C). Utilising Hi-C data [19] we observed an interaction between the CpGs and a region encompassing the 5′ end of *SLBP*, the entirety of *TMEM129*, and the 5′ end of *TACC3* in IMR-90 cells, a lung fibroblast cell line, and in H1-hESC cells, an embryonic stem cell line (Fig. 1C). These interactions were only observed in these cell lines.

### Methylation QTL analysis of rs11732213

We next replicated the mQTL analysis of cg20987369 and cg25007799 that we had previously reported [6] in an independent cohort of patient cartilage DNA samples and expanded the analysis to encompass additional CpGs, which flanked and fell between cg20987369 and cg25007799 (Fig. 1D and E). In total, we analysed 14 CpGs, numbered in order of their physical location, which spanned 259bp. The discovery CpGs, cg20987369 and cg25007799, are numbered CpG8 and CpG13, respectively. Nine of the CpGs showed a significant (*P* < 0.05) correlation between DNA methylation levels and rs11732213 genotype, confirming that this is a differentially methylated region (DMR) in cartilage (Fig. 1E and Supplementary Table 5). The OA risk-conferring T allele of rs11732213 correlated with lower methylation, as we had previously observed for the discovery CpGs cg20987369 and cg25007799 in the 450K array data [6].

**Fig. 2:**
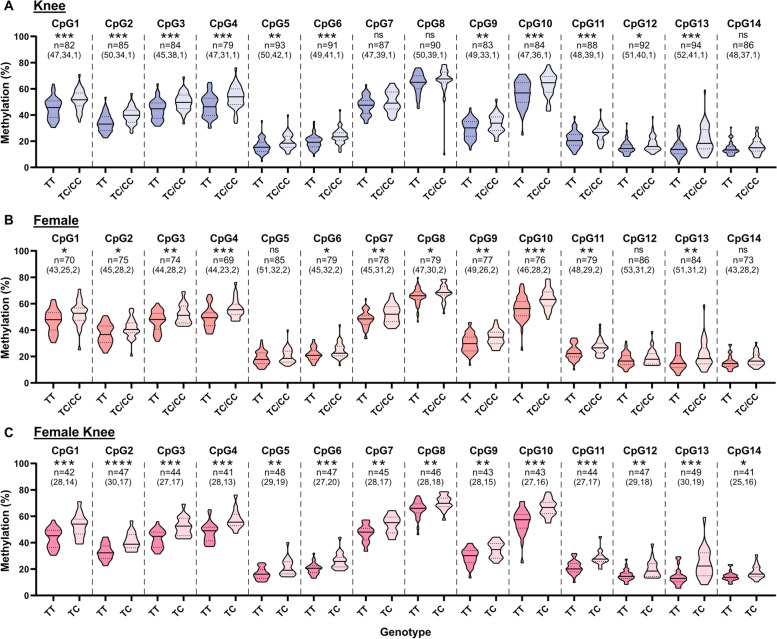
The mQTL is particularly pronounced in females with knee OA. Data was stratified into knee (**A**), female (**B**) and female knee (**C**) strata. The methylation data is in the form of *β*-values ranging from 0 (no methylation) to 1 (complete methylation) and expressed as a percentage. Due to their low number (< 3) in each stratum, minor allele homozygotes (CC) were combined with heterozygotes (TC). There were no minor allele homozygotes in the female knee strata. In the violin plots, solid and dashed horizontal lines represent the median and interquartile range. Difference in numbers (*n*) due to variable number of patient samples per CpG passing quality control, with numbers in parentheses the number of patients per genotype (TT, TC, CC). *P*-values calculated by linear regression. * = *P* < 0.05; ** = *P* < 0.01; *** = *P* < 0.001; **** = *P* < 0.0001; ns = not significant (*P* > 0.05)

When methylation data was stratified irrespective of genotype, DNA methylation at six of the CpGs (CpGs 1, 2, 3, 5, 12 and 14) was found to be significantly higher in hip compared to knee cartilage (Supplementary Fig. 1A). In contrast CpGs 8 and 10 exhibited significantly lower DNA methylation in hip compared to knee (Supplementary Fig. 1A). Stratification by sex showed significantly increased levels of methylation in females compared to males at CpGs 4, 7, 8 and 9 (Supplementary Fig. 1B). We therefore plotted the mQTL data following stratification by joint, by sex, and by joint and sex (Fig. 2 and Supplementary Fig. 2). In the knee, the female, and the female-knee strata, the majority, or all, of the CpGs demonstrated a significant mQTL (Fig. 2).

### The DMR is a methylation-sensitive enhancer

**Fig. 3:**
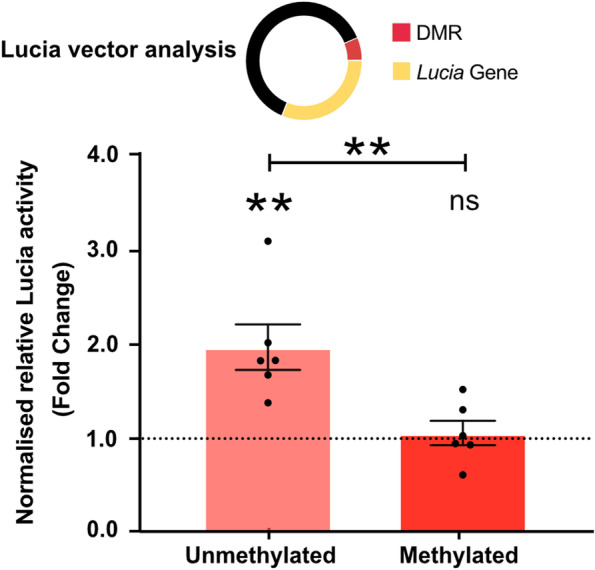
Investigation of enhancer activity at the DMR in Tc28a2 chondrocytes. Top, cartoon of vector showing DMR preceding Lucia gene. Bottom, Lucia reporter assays assessing enhancer activity in the presence of construct containing the DMR in an unmethylated or methylated state. Values were normalised to those in empty vector control (dotted horizontal line). Black dots represent individual samples (*n* = 6 replicates per group). Bars show the mean ± SEM. *P*-values calculated by unpaired *t*-test

We cloned a 568bp region encompassing CpGs 1-14 into a CpG-free Lucia reporter vector and tested the DMR for enhancer activity and methylation sensitivity in Tc28a2 immortalised chondrocytes (Fig. 3 and Supplementary Table 6). The unmethylated region acted as an enhancer, exhibiting a 1.9-fold increase in reporter gene expression compared to the control vector lacking the DMR sequence (*P* < 0.001). Methylation of the region abolished this enhancer effect, with expression being no different to control vector.

### DMR demethylation increases *TMEM129* and *SLBP* expression

**Fig. 4:**
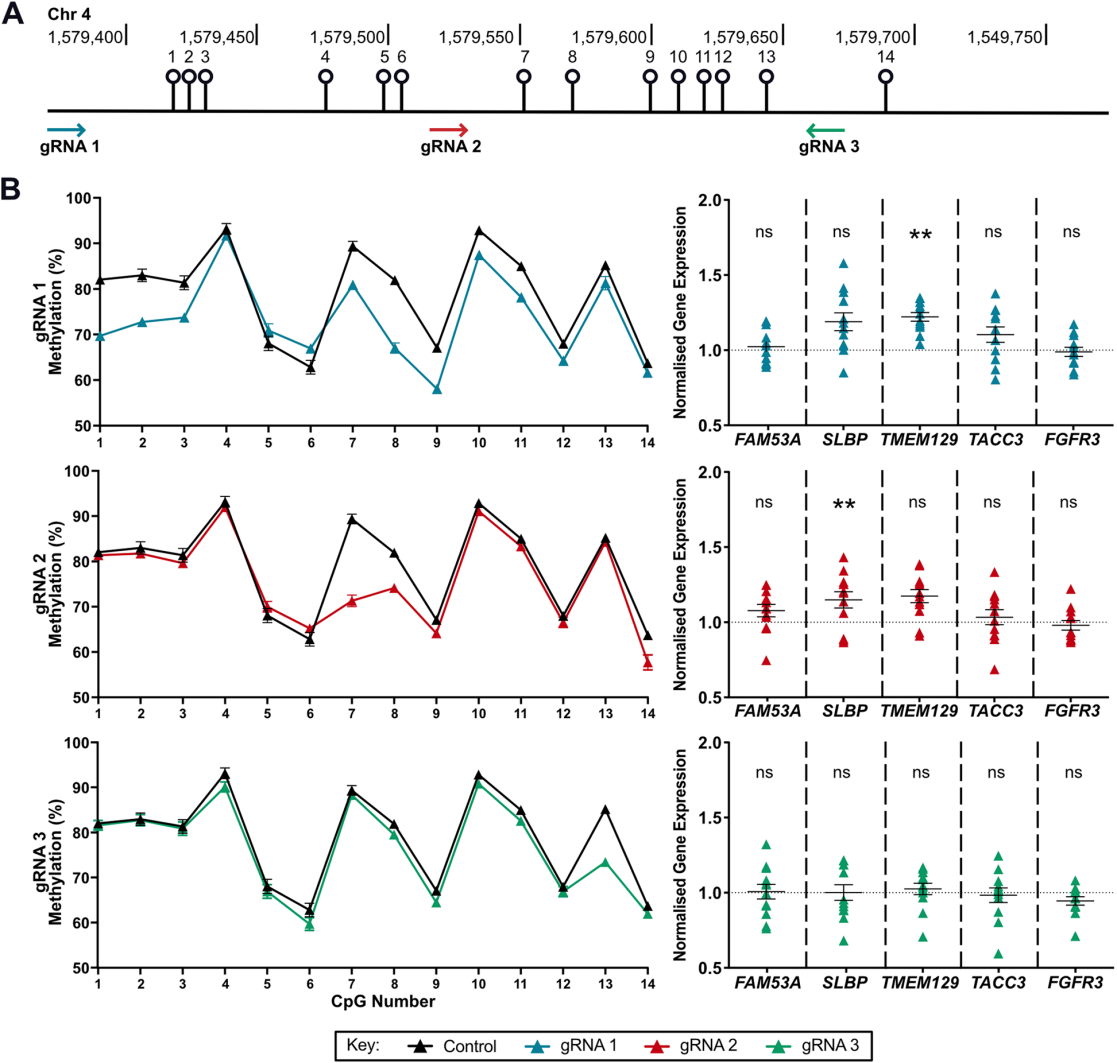
Epigenome modulation of the DMR in Tc28a2 chondrocytes. **A** Schematic diagram showing the position of gRNAs 1, 2 and 3 used for demethylation, relative to the 14 CpGs. **B** Left, DNA methylation levels of the 14 CpGs following expression of dCas9-TET1 protein in control (black line), with non-targeting gRNA, or in samples with a targeting gRNA (coloured lines). Twelve replicates for control and for each targeting gRNA. Bars show the mean ± SEM. Right, *FAM53A*, *SLBP*, *TMEM129*, *TACC3* and *FGFR3* expression following editing of DNA methylation with gRNAs. Values were normalised to those of control (dotted horizontal line). Triangles represent individual samples (*n* = 12 replicates). Bars show the mean ± SEM. *P*-values calculated by multiple paired *t*-tests with Holm-Šídák approach to account for multiple comparisons. ** = *P* < 0.01; ns = not significant (*P* > 0.05)

We next investigated the effect of altering the DNA methylation status of the DMR in Tc28a2 chondrocytes upon target gene expression. Tc28a2 cells are heterozygous TC at rs11732213 and in this cell line, the 14 CpGs are hypermethylated, with DNA methylation levels ranging from 60% to > 90% (Fig. 4B, left section, control data, black lines). We therefore demethylated the DMR using dCas9-TET1. Three gRNAs (gRNAs 1–3) were employed (Fig. 4A) and a reduction in DNA methylation at the DMR was observed with each gRNA, with gRNA1 inducing a reduction across most CpGs (Fig. 4B and Supplementary Table 7). The largest reduction per gRNA was 15% at CpG8 for gRNA1, 18% at CpG7 for gRNA2 and 12% at CpG13 for gRNA3.

We measured the effect of DMR demethylation on the expression of the five putative effector genes at the locus that are expressed in both human articular cartilage [6] and Tc28a2 cells: *FAM53A*, *SLBP*, *TMEM129*, *TACC3* and *FGFR3*. The gRNA1 demethylation resulted in a 1.22-fold increase in *TMEM129* expression (*P* = 0.0039) whilst the gRNA2 demethylation resulted in a 1.15-fold increase in *SLBP* expression (*P* = 0.0025) (Fig. 4B). No other significant changes in expression were observed.

### The DMR is predicted to bind transcription factors that are expressed in cartilage

**Fig. 5:**
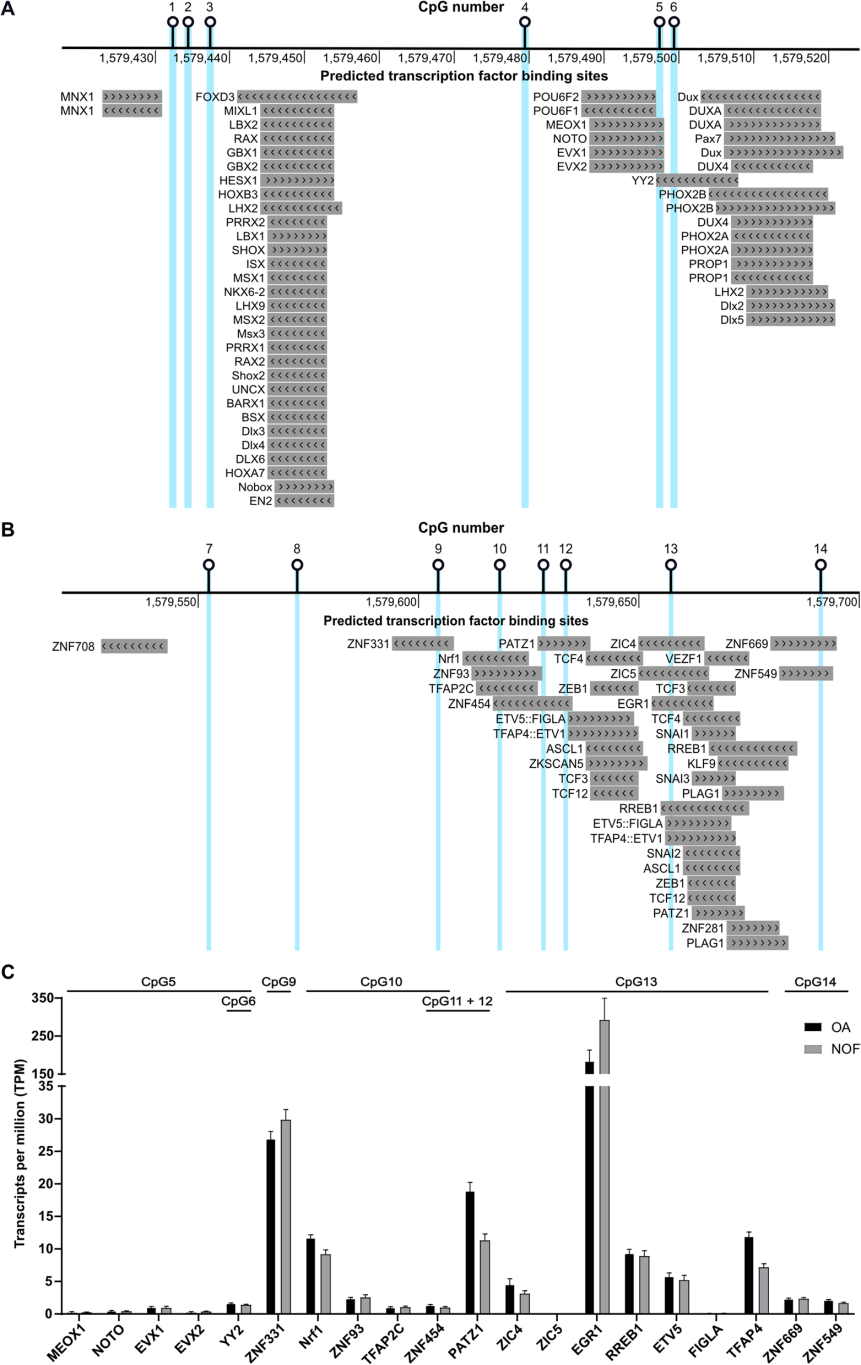
Transcription factors (TFs) predicted to bind at or close to DMR CpGs. **A**, **B** TF binding sites within 10bp of each DMR CpG as predicted by JASPAR, visualised in the UCSC Genome Browser (hg19). The TFs are marked by grey bars with the direction of the arrows within the boxes indicating the DNA strand the TF is predicted to bind to (arrows pointing to the left = antisense strand; arrows pointing to the right = sense strand). **C** Expression levels (TPM, transcripts per million) of the TFs predicted to bind at CpGs in hip cartilage RNA-sequencing data from OA (*n* = 10, black) and NOF (*n* = 6, grey) patients. The bars represent the mean and the SEM. The *y*-axis is a linear segmented scale with two segments

The Lucia reporter and dCas9 experiments imply that changing the DMR methylation status has direct effects on the functioning of the enhancer and on gene expression. A potential mechanism for this could be an alteration of the binding efficiency of transcription factors to DNA that can occur in response to methylation changes [7, 8]. If this were to be the mechanism by which the DMR regulates enhancer activity, we would expect the CpGs to be part of, or physically close to, transcription factor binding sites. To assess this, we used JASPAR [20] and identified transcription factors predicted to bind at or near DMR CpGs (Fig. 5A and B). Several of those predicted to bind at CpGs are expressed in cartilage (Fig. 5C). *EGR1*, predicted to bind at cg25007799 (CpG13, Fig. 5B), was the most abundantly expressed, with transcripts per million (TPM) values > 150 (Fig. 5C).

### TMEM129 shows allelic expression imbalance associating with OA risk

The epigenome editing highlighted *TMEM129* and *SLBP* as target genes of the DMR. If, as we hypothesised, there is a functional link between rs11732213 genotype, the DMR and target gene expression, then we would expect the expression of one, or both, of these genes to also correlate with genotype at rs11732213. To assess this, we undertook allelic expression imbalance (AEI) analysis in patient cartilage samples. rs11732213 is located within intron 3 of *SLBP*, so we first identified transcript SNPs for *TMEM129* and *SLBP*. For *TMEM129* we chose rs2236786 (T > C), which is in the penultimate exon of the gene. This SNP is in near perfect linkage disequilibrium (LD) with rs11732213 (*r*^2^ = 0.97, D′ = 1), with the OA risk-conferring allele T of rs11732213 correlating with allele T of rs2236786 (T-T haplotype). For *SLBP*, we chose rs2247341 (G > A), which is in the fifth exon of the gene. rs11732213 and rs2247341 are in complete LD (*r*^2^ = 0.14 but D′ = 1), with allele A of rs2247341 always occurring on a chromosome containing allele T at rs11732213 (T-A haplotype).

**Fig. 6:**
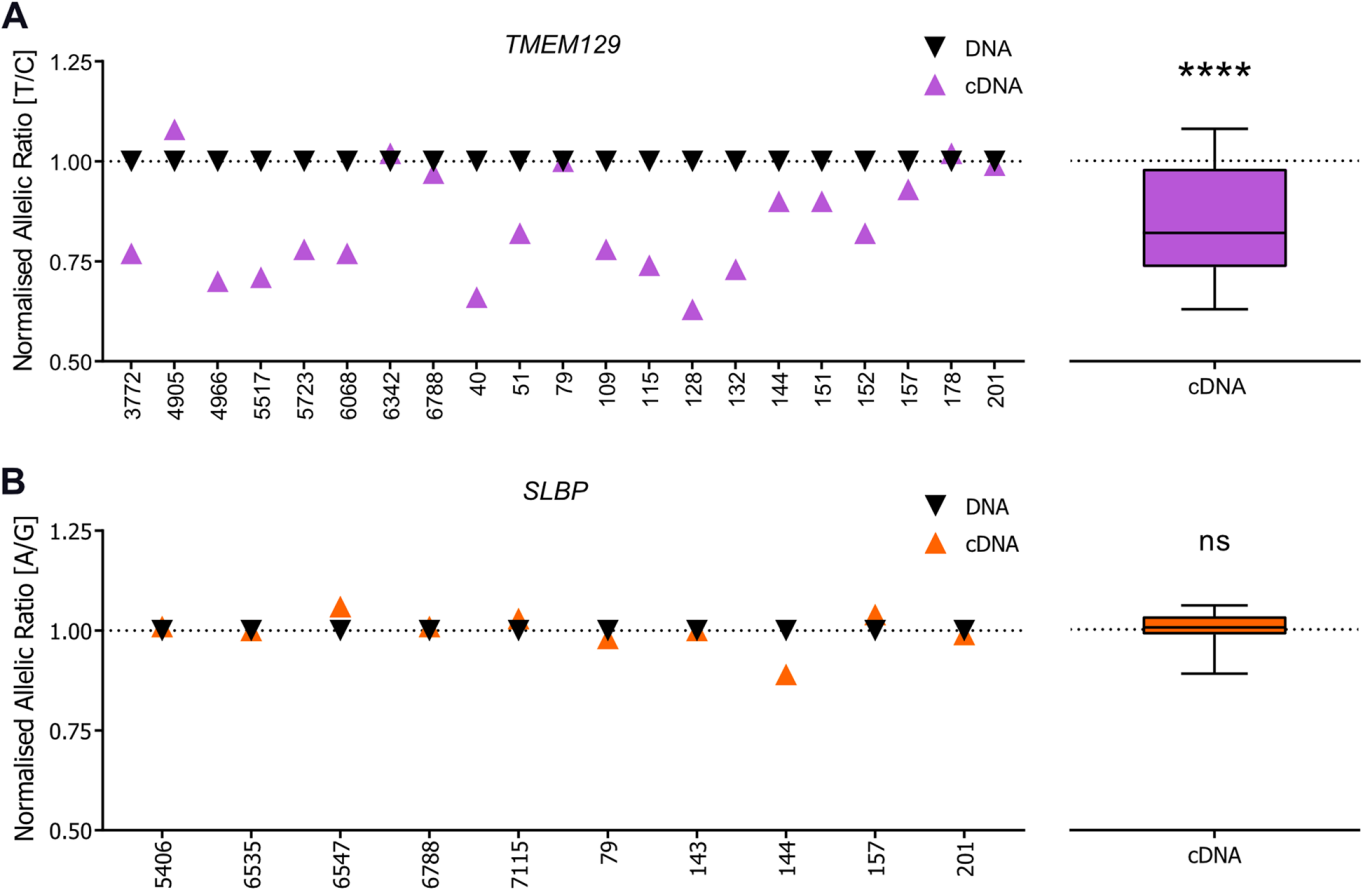
Allelic expression imbalance (AEI) analysis of *TMEM129* and *SLBP*. **A** Left, allelic (T/C) ratios in cartilage samples from OA patients heterozygous for *TMEM129* transcript SNP rs2236786 (*n* = 21, numbers on *x*-axis are patient sample IDs). In each sample, the ratio of values for cDNA and DNA between the OA risk-conferring allele T and allele C was plotted; each triangle represents the mean of three technical repeats (purple, cDNA; black, DNA). Right, mean cDNA values for all samples represented as a box plot normalised to their corresponding DNA values (dotted line), with the line inside the box representing the median, the box the interquartile range, and whiskers showing the minimum and maximum values. **B** Left, allelic (A/G) ratios in cartilage samples from OA patients heterozygous for *SLBP* transcript SNP rs2247341 (*n* = 10). In each sample, the ratio of values for cDNA and DNA between the OA risk-conferring allele A and allele G was plotted; each triangle represents the mean of three technical repeats (orange, cDNA; black, DNA). Right, mean cDNA values for all samples represented as a box plot normalised to their corresponding DNA values, as above. *P*-values calculated by the Wilcoxon matched-pairs signed rank test. **** = *P* < 0.0001; ns = not significant (*P* > 0.05)

For each gene, we analysed patients compound heterozygous at rs11732213 and the transcript SNP. We analysed 21 compound heterozygotes for *TMEM129* and 10 compound heterozygotes for *SLBP*. The larger number of compound heterozygotes studied for *TMEM129* reflects the higher frequency of the T-T (rs11732213-rs2236786) haplotype compared to the T-A (rs11732213-rs2247341) haplotype; 78% versus 33% in European ancestry cohorts. Highly significant AEI was observed at *TMEM129* (*P* < 0.0001; Fig. 6A and Supplementary Table 8), with a mean T/C ratio of 0.84. The OA risk-conferring allele T of rs11732213 corresponded with reduced expression of the gene. We did not detect AEI at *SLBP* (*P* > 0.05; Fig. 6B).

## Discussion

We and others have shown that OA association SNPs often correlate with the expression level of genes in relevant tissues, such as cartilage, and that incorporating epigenetics into the analysis of these signals provides enriched mechanistic insight by highlighting epigenetics as an intermediate between risk alleles and effector genes [6, 13–16, 23, 25–27]. In this study, we investigated the OA signal marked by SNP rs11732213, which is associated with hip and knee OA [2]. Since genotype at rs11732213 correlated with cartilage DNA methylation at two nearby CpGs, forming an mQTL [6], we hypothesised that this signal is one in which epigenetic effects are functionally relevant to increased OA risk.

We replicated the cartilage rs11732213 mQTL and demonstrated that it extended beyond the two discovery CpGs present on the 450K array, forming a DMR spanning over 250bp. The DMR was more pronounced in female knee OA samples. Sex-related differences in DNA methylation have been reported [28, 29] as have differences between hip and knee cartilage [30, 31]. Although the GWAS that discovered the rs11732213 OA locus reported it as a knee and a hip signal, and not specific to one sex [2], our data implies that the epigenetic functional effect at this locus is particularly pronounced in females with knee OA. This subgroup should therefore be the focus of further investigations of this association signal.

We next undertook in vitro experiments on the DMR using the human chondrocyte cell line Tc28a2. Cloning the DMR into a reporter construct demonstrated that it acts as an enhancer in an unmethylated state. When we demethylated the DMR using dCas9-TET1, we observed increased expression of *TMEM129* and *SLBP*. An additional link between the DMR and *TMEM129* came from our in silico analysis, which identified physical interactions between the CpGs and *TMEM129*, implying that the DMR can regulate expression of the gene. A search of JASPAR identified transcription factors predicted to bind within the DMR, several of which are expressed in cartilage. Of note is *EGR1*, coding for the zinc-finger transcription factor early growth response protein 1. This transcription factor has been reported as having enriched binding sites within, and to be a regulator of, genes that show differential expression between OA and non-OA cartilage [32]. It is predicted to bind across cg25007799, one of the two discovery CpGs for the locus, and in our cartilage RNA-sequencing analysis, the *EGR1* gene was abundantly expressed. The EGR1 transcription factor, and others predicted to bind within the DMR, could be functional links between the DMR and target gene expression. They merit targeted experimental investigation, initially using approaches such as electrophoretic mobility shifts assays (EMSAs) and chromatin immunoprecipitation (ChIP) to confirm transcription factor binding at the CpG sites.

For *TMEM129*, we subsequently demonstrated that the OA risk-conferring allele T of rs11732213 is expressed in hip and knee articular cartilage at a lower level than its non-risk allele C. In this tissue, allele T corresponded with reduced DNA methylation at the DMR CpGs. Reduced methylation therefore correlates with reduced *TMEM129* expression. However, in our Tc28a2 dCas9-TET1 experiment, reduced DNA methylation led to increased *TMEM129* expression. This highlights the need to combine *in vitro* experiments, which are models, with an analysis of patient cells, which are pathologically relevant to the disease, to draw appropriate conclusions regarding genetic and epigenetic effects on disease risk.

Our investigations have highlighted *TMEM129* as an effector gene of the rs11732213 OA association signal. What our data does not do is exclude the possibility that this signal impacts on the activity of other genes at this locus; it is possible that there are multiple effector genes, with our experiments highlighting the role of one of these.

*TMEM129* encodes the protein E3 ubiquitin-protein ligase TM129. This enzyme is localised to the endoplasmic reticulum (ER) where it is involved in protein degradation through regulated proteolysis and through the unfolded protein response (UPR) [33]. Genetic analysis of monogenic diseases has mechanistically linked the UPR and ER-stress to rare chondrodysplasias that have early onset secondary OA as one of their phenotypic components [34], whilst analysis of chondrocyte and cartilage biology data has highlighted a role for impaired UPR in primary OA [34, 35]. Our results now functionally link a common OA genetic association signal to this important homeostatic pathway. We propose that the risk conferring allele at the association signal alters the methylation status of the DMR leading to reduced expression of *TMEM129* and lower levels of its encoded protein, which has a detrimental impact on the functioning of the UPR in chondrocytes, increasing disease risk. The DMR, *TMEM129* and the UPR should now be prioritised for translational follow-up as potential treatments for OA.

## Conclusions

The functional investigation of loci encoding genetic risk for complex diseases requires a combination of statistical fine-mapping, *in silico* analyses, and laboratory-based experimental approaches [36, 37]. In this report, we primarily focussed on laboratory studies to characterise the OA association signal marked by SNP rs11732213. Our data suggests that *TMEM129* is a target of this signal. The GWAS that discovered the signal used in silico analysis (primarily eQTL data from the Genotype-Tissue Expression (GTEx) portal) to highlight *FGFR3* as a potential target [2]. A subsequent larger GWAS, that included UK Biobank and arcOGEN data alongside additional cohorts, reported the same signal, albeit with a different lead SNP, rs1530586 (r^2^ of 0.89 with rs11732213 in European ancestry cohorts) [38]. In silico analysis in this larger GWAS again highlighted *FGFR3* [38]. Our study emphasises the importance of detailed laboratory-based functional studies to help identify OA causal genes and, in particular, the use of disease-relevant tissues, cells, and experimental models. Without these, investigators could mistakenly overlook genes or pathways that are not prioritised by current literature.

## Supplementary Information

**Additional file 1: Supplementary Table 1.** Details of the 165 osteoarthritis patients included in this study.

**Additional file 2: Supplementary Table 2.** Primers used in this study. [Btn], biotin tag at the 5' end of the primer. n/a, not applicable. Lower case sequence represents restriction enzyme motifs for cloning applications: cctagg, *AvrII*; actagt, *SpeI*.

**Additional file 3: Supplementary Table 3.** The sequences of guide RNAs (gRNAs) used for dCas9-TET1 modulation of the epigenome.

**Additional file 4: Supplementary Table 4.** Details of the pre-designed RT-qPCR assays used to quantify gene expression.

**Additional file 5: Supplementary Table 5.** Methylation data (β-values expressed as a percentage) used for mQTL analysis. *P*-values calculated by linear regression.

**Additional file 6: Supplementary Fig. 1.** DNA methylation data stratified by joint (A) and by sex (B) irrespective of rs11732213 genotype. Methylation data is in the form of *β*-values ranging from 0 (no methylation) to 1 (complete methylation) and expressed as a percentage. In the violin plots, solid and dashed horizontal lines represent the median and interquartile range. Difference in numbers (*n*) due to variable number of patient samples per CpG passing quality control. *P*-values calculated by Mann-Whitney *U* test. * = *P* < 0.05; ** = *P* < 0.01; *** = *P* < 0.001; **** = *P* < 0.0001; ns = not significant (*P* > 0.05).

**Additional file 7: Supplementary Fig. 2.** mQTL plots stratified into hip (A), male (B), female hip (C), male hip (D) and male knee (E) strata. The methylation data is in the form of *β*-values ranging from 0 (no methylation) to 1 (complete methylation) and expressed as a percentage. Due to their low number (< 3) in each stratum, minor allele homozygotes (CC) were combined with heterozygotes (TC). There were no minor allele homozygotes in the male hip strata. In the violin plots, solid and dashed horizontal lines represent the median and interquartile range. Difference in numbers (n) due to variable number of patient samples per CpG passing quality control, with numbers in parentheses the number of patients per genotype (TT, TC, CC). *P*-values calculated by linear regression. * = *P* < 0.05; ns = not significant (*P* > 0.05).

**Additional file 8: Supplementary Table 6.** Lucia reporter analysis data. *P*-values calculated using unpaired *t*-test.

**Additional file 9: Supplementary Table 7.** Data from the dCas9-TET1 experiment. *P*-values calculated using multiple paired *t*-tests with Holm-Šídák approach to account for multiple comparisons.

**Additional file 10: Supplementary Table 8.** Data from the allelic expression imbalance (AEI) analysis of *TMEM129* and *SLBP*. *P*-values calculated using Wilcoxon matched-pairs signed rank test.

## Data Availability

Raw data is presented in Additional files 5, 8, 9 and 10 (Supplementary Tables 5-8).

## Abbreviations

AEI: Allelic expression imbalance
ChIA-PET: Chromatin Interaction Analysis by Paired-End Taq Sequencing
ChIP: Chromatin immunoprecipitation
CpG: Cytosine-Guanine dinucleotide
CRISPR: Clustered regularly interspaced short palindromic repeats
DMR: Differentially methylated region
EMSA: Electrophoretic mobility shifts assay
ER: Endoplasmic reticulum
gRNA: Guide RNA
GEO: Gene Expression Omnibus
GTEx: Genotype-Tissue Expression
GWAS: Genome-wide association study
Hi-C: High-throughput chromatin conformation capture
LD: Linkage disequilibrium
mQTL: Methylation quantitative trait locus
NHS: National Health Service
NOF: Neck of femur
OA: Osteoarthritis
RT-qPCR: Real-time quantitative polymerase chain reaction
SEM: Standard error of mean
SNP: Single nucleotide polymorphism
TPM: Transcripts per million
UCSC: University of California Santa Cruz
UPR: Unfolded protein response

## References

[1] Aubourg G, Rice SJ, Bruce-Wootton P, Loughlin J (2022). Genetics of osteoarthritis. Osteoarthritis Cartilage..

[2] Tachmazidou I, Hatzikotoulas K, Southam L, Esparza-Gordillo J, Haberland V, Zheng J (2019). Identification of new therapeutic targets for osteoarthritis through genome-wide analyses of UK Biobank data. Nat Genet..

[3] Gallagher MD, Chen-Plotkin AS (2018). The post-GWAS era: from association to function. Am J Hum Genet..

[4] Smith E, Shilatifard A (2014). Enhancer biology and enhanceropathies. Nat Struct Mol Biol..

[5] Boix CA, James BT, Park YP, Meuleman W, Kellis M (2021). Regulatory genomic circuitry of human disease loci by integrative epigenomics. Nature..

[6] Rice SJ, Cheung K, Reynard LN, Loughlin J (2019). Discovery and analysis of methylation quantitative trait loci (mQTLs) mapping to novel osteoarthritis genetic risk signals. Osteoarthritis Cartilage..

[7] Zhu H, Wang G, Qian J (2016). Transcription factors as readers and effectors of DNA methylation. Nat Rev Genet..

[8] Héberlé E, Bardet AF (2019). Sensitivity of transcription factors to DNA methylation. Essays Biochem..

[9] Hannon E, Gorrie-Stone TJ, Smart MC, Burrage J, Hughes A, Bao Y (2018). Leveraging DNA-methylation quantitative-trait loci to characterize the relationship between methylomic variation, gene expression, and complex traits. Am J Hum Genet..

[10] Pierce BL, Tong L, Argos M, Demanelis K, Jasmine F, Rakibuz-Zaman M (2018). Co-occurring expression and methylation QTLs allow detection of common causal variants and shared biological mechanisms. Nat Commun..

[11] Villicaña S, Bell JT (2021). Genetic effects on DNA methylation: research findings and future perspectives. Genome Biol..

[12] Adkar SS, Brunger JM, Willard VP, Wu C-L, Gersbach CA, Guilak F (2017). Genome engineering for personalized arthritis therapeutics. Trends Mol Med..

[13] Rice SJ, Beier F, Young DA, Loughlin J (2020). Interplay between genetics and epigenetics in osteoarthritis. Nat Rev Rheumatol..

[14] Parker E, Hofer IM, Rice SJ, Earl L, Anjum S, Deehan D (2021). Multi-tissue epigenetic and gene expression analysis combined with epigenome modulation identifies RWDD2B as a target of osteoarthritis susceptibility. Arthritis Rheumatol..

[15] Rice SJ, Roberts JB, Tselepi M, Brumwell A, Falk J, Steven C (2021). Genetic and epigenetic fine-tuning of TGFB1 expression within the human osteoarthritic joint. Arthritis Rheumatol..

[16] Kehayova YS, Watson E, Wilkinson JM, Loughlin J, Rice SJ (2021). Genetic and epigenetic interplay within a COLGALT2 enhancer associated with osteoarthritis. Arthritis Rheumatol..

[17] Kent W, Sugnet C, Furey T, Roskin K, Pringle T, Zahler A (2002). The human genome browser at UCSC. Genome Res..

[18] Roadmap Epigenomics Consortium (2015). Integrative analysis of 111 reference human epigenomes. Nature..

[19] Zhou X, Maricque B, Xie M, Li D, Sundaram V, Martin EA (2011). The Human Epigenome Browser at Washington University. Nat Methods..

[20] Castro-Mondragon JA, Riudavets-Puig R, Rauluseviciute I, Lemma RB, Turchi L, Blanc-Mathieu R (2022). JASPAR 2022: the 9th release of the open-access database of transcription factor binding profiles. Nucleic Acids Res..

[21] Ajekigbe B, Cheung K, Xu Y, Skelton AJ, Panagiotopoulos A, Soul J (2019). Identification of long non-coding RNAs expressed in knee and hip osteoarthritic cartilage. Osteoarthritis Cartilage..

[22] Kokenyesi R, Tan L, Robbins JR, Goldring MB (2000). Proteoglycan production by immortalized human chondrocyte cell lines cultured under conditions that promote expression of the differentiated phenotype. Arch Biochem Biophys..

[23] Rice S, Aubourg G, Sorial A, Almarza D, Tselepi M, Deehan D (2018). Identification of a novel, methylation-dependent, RUNX2 regulatory region associated with osteoarthritis risk. Hum Mol Genet..

[24] Du P, Zhang X, Huang C-C, Jafari N, Kibbe WA, Hou L (2010). Comparison of Beta-value and M-value methods for quantifying methylation levels by microarray analysis. BMC Bioinformatics..

[25] Steinberg J, Ritchie GRS, Roumeliotis TI, Jayasuriya RL, Clark MJ, Brooks RA (2017). Integrative epigenomics, transcriptomics and proteomics of patient chondrocytes reveal genes and pathways involved in osteoarthritis. Sci Rep..

[26] Steinberg J, Southam L, Roumeliotis TI, Clark MJ, Jayasuriya RL, Swift D (2021). A molecular quantitative trait locus map for osteoarthritis. Nat Commun..

[27] Liu Y, Chang J-C, Hon C-C, Fukui N, Tanaka N, Zhang Z (2018). Chromatin accessibility landscape of articular knee cartilage reveals aberrant enhancer regulation on osteoarthritis. Sci Rep..

[28] van Dongen J, Nivard MG, Willemsen G, Hottenga J-J, Helmer Q, Dolan CV (2016). Genetic and environmental influences interact with age and sex in shaping the human methylome. Nat Commun..

[29] Lin X, Li L, Liu X, Tian J, Zheng W, Li J (2020). Genome-wide analysis of aberrant methylation of enhancer DNA in human osteoarthritis. BMC Med Genomics..

[30] den Hollander W, Ramos YF, Bos SD, Bomer N, van der Breggen R, Lakenberg N (2014). Knee and hip articular cartilage have distinct epigenomic landscapes: implications for future cartilage regeneration approaches. Ann Rheum Dis..

[31] Rushton MD, Reynard LN, Barter MJ, Refaie R, Rankin KS, Young DA (2014). Characterization of the cartilage DNA methylome in knee and hip osteoarthritis. Arthritis Rheumatol..

[32] Fisch KM, Gamini R, Alvarez-Garcia O, Akagi R, Saito M, Muramatsu Y (2018). Identification of transcription factors responsible for dysregulated networks in human osteoarthritis cartilage by global gene expression analysis. Osteoarthritis Cartilage..

[33] van de Weijer ML, Bassik MC, Luteijn RD, Voorburg CM, Lohuis MAM, Kremmer E (2014). A high-coverage shRNA screen identifies TMEM129 as an E3 ligase involved in ER-associated protein degradation. Nat Commun..

[34] Briggs MD, Dennis EP, Dietmar HF, Pirog KA (2020). New developments in chondrocyte ER stress and related diseases. F1000Res.

[35] Rellmann Y, Eidhof E, Dreier R (2021). Review: ER stress-induced cell death in osteoarthritic cartilage. Cell Signal..

[36] Lichou F, Trynka G (2020). Functional studies of GWAS variants are gaining momentum. Nat Commun..

[37] Lappalainen T, MacArthur DG (2021). From variant to function in human disease genetics. Science..

[38] Boer CG, Hatzikotoulas K, Southam L, Stefánsdóttir L, Zhang Y, Coutinho de Almeida R (2021). Deciphering osteoarthritis genetics across 826,690 individuals from 9 populations. Cell..

