# Supplementary figures and images for: Identification of TMEM129, encoding a ubiquitin-protein ligase, as an effector gene of osteoarthritis genetic risk
Source: Arthritis Res Ther. 2022 Aug 8;24:189. doi: 10.1186/s13075-022-02882-y (PMC9358880; doi:10.1186/s13075-022-02882-y)

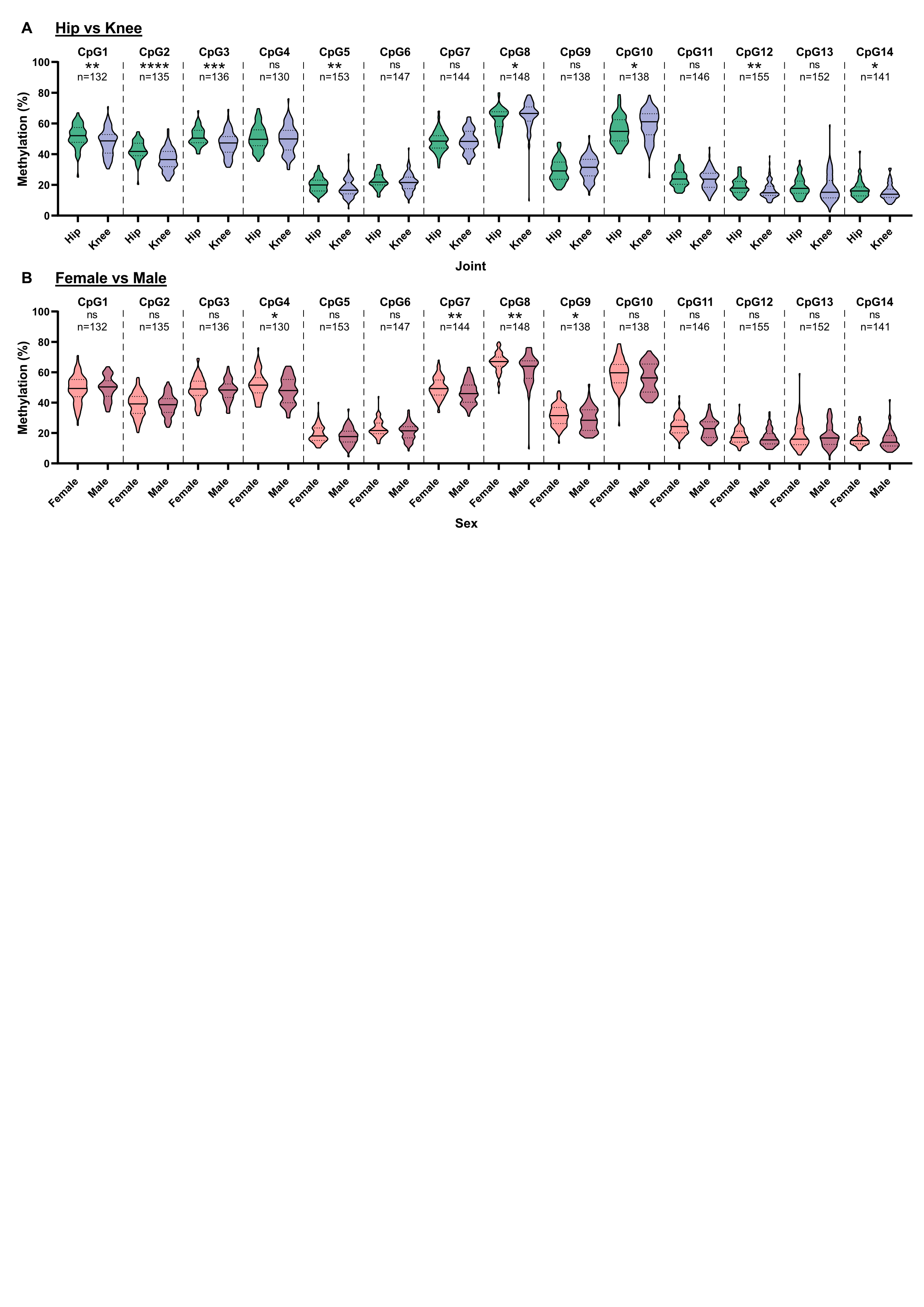

Supplement: Supplementary file 6 — Additional file 6: Supplementary Fig. 1. DNA methylation data stratified by joint (A) and by sex (B) irrespective of rs11732213 genotype. Methylation data is in the form of β-values ranging from 0 (no methylation) to 1 (complete methylation) and expressed as a percentage. In the violin plots, solid and dashed horizontal lines represent the median and interquartile range. Difference in numbers (n) due to variable number of patient samples per CpG passing quality control. P-values calculated by Mann-Whitney U test. * = P < 0.05; ** = P < 0.01; *** = P < 0.001; **** = P < 0.0001; ns = not significant (P > 0.05). [file 13075_2022_2882_MOESM6_ESM.tiff]

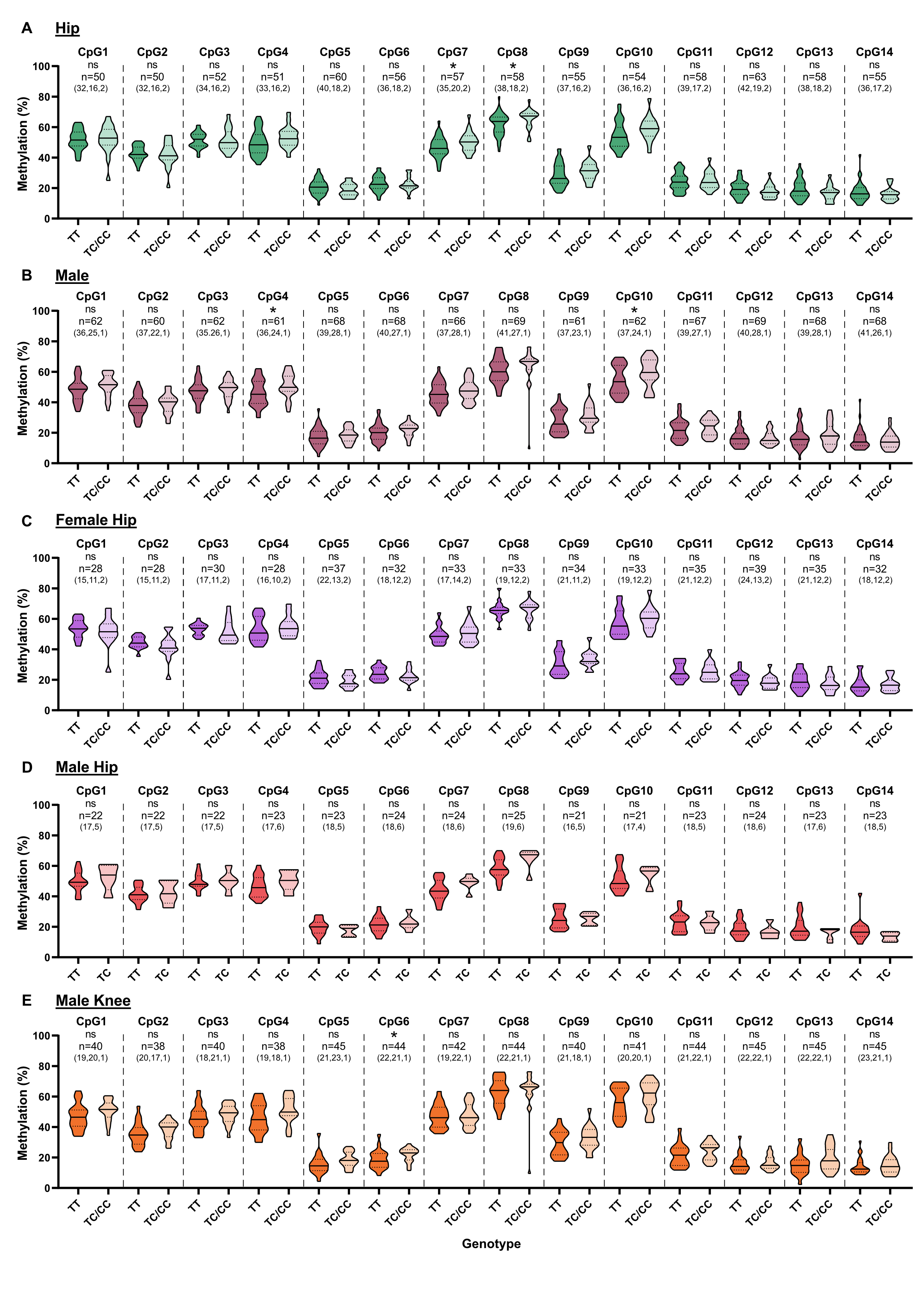

Supplement: Supplementary file 7 — Additional file 7: Supplementary Fig. 2. mQTL plots stratified into hip (A), male (B), female hip (C), male hip (D) and male knee (E) strata. The methylation data is in the form of β-values ranging from 0 (no methylation) to 1 (complete methylation) and expressed as a percentage. Due to their low number (< 3) in each stratum, minor allele homozygotes (CC) were combined with heterozygotes (TC). There were no minor allele homozygotes in the male hip strata. In the violin plots, solid and dashed horizontal lines represent the median and interquartile range. Difference in numbers (n) due to variable number of patient samples per CpG passing quality control, with numbers in parentheses the number of patients per genotype (TT, TC, CC). P-values calculated by linear regression. * = P < 0.05; ns = not significant (P > 0.05). [file 13075_2022_2882_MOESM7_ESM.tiff]
